# The efficacy of olaparib as salvage therapy in an advanced intrahepatic cholangiocarcinoma patient harboring somatic *BRCA1* and *PALB2* pathogenic variants: a case report and literature review

**DOI:** 10.3389/fphar.2025.1558677

**Published:** 2025-08-01

**Authors:** Jian Wang, Qinhong Zheng, Jianxin Chen

**Affiliations:** Department of Medical Oncology, The Quzhou Affiliated Hospital of Wenzhou Medical University, Quzhou People’s Hospital, Quzhou, Zhejiang, China

**Keywords:** *BRCA1*, *PALB2*, olaparib, intrahepatic cholangiocarcinoma, efficacy, case report, literature review

## Abstract

**Background:**

For advanced biliary tract cancer (BTC) patients with *BRCA* pathogenic variants who have failed first-line treatment, the optimal treatment strategy remains to be established. Olaparib, the first FDA-approved poly adenosine diphosphate-ribose polymerase inhibitors (PARPi), is commonly utilized in clinical practice for breast, ovarian, prostate, and pancreatic cancers that harbor germline or somatic *BRCA* pathogenic variants through a mechanism known as “synthetic lethality”. However, the proportion of BTC patients with *BRCA* pathogenic variants is relatively low, estimated at approximately 1%–7% of all BTC cases, leading to inconclusive evidence regarding the efficacy of targeted therapy with PARPi for these patients.

**Case presentation:**

We presented a case of a patient with advanced intrahepatic cholangiocarcinoma (iCCA) harboring dual somatic homologous recombination repair (HRR) gene pathogenic variants, specifically *BRCA1* and *PALB2*, who achieved PR lasting approximately 7 months following salvage treatment with olaparib.

**Conclusion:**

We considered that the BTC population with dual HRR pathogenic variants, which include a *BRCA* pathogenic variant, might represent an advantageous cohort for olaparib treatment. Furthermore, in addition to *BRCA* pathogenic variant, *PALB2* pathogenic variant may potentially serve as the next clinical predictive target for PARP inhibitors in the BTC population. A systematic summary and analysis of existing studies on BTC patients with pathogenic variants indicate that these patients might derive benefits from olaparib; however, further validation in a larger cohort is necessary.

## Highlights

• BRCA1 and PALB2 double HRR gene mutations *may be associated with* an increased risk of resistance to chemotherapy and immune checkpoint inhibitors in ICCA patients, while *potentially enhancing* sensitivity to PARP inhibitor (PARPi) treatment.
• BTC patients with BRCA1/2 mutations *may derive clinical benefit* from PARPi therapy, and those harboring dual HRR gene mutations (including one BRCA variant) *might represent* a subgroup *worthy of further investigation* as potential beneficiaries of PARPi.
• These findings *underscore the possible importance* of individualized treatment strategies guided by molecular profiling in the second-line setting. Furthermore, PALB2 mutations *may emerge as* a promising predictive biomarker following BRCA in the context of BTC, *pending further validation in larger cohorts*.

## Introduction

Biliary tract cancer (BTC) is the second most common primary liver tumor (Vogel et al., 2023). Due to its highly invasive, occult, and heterogeneous nature, the prognosis is very poor, with a five-year survival rate of approximately 5% (Zhang et al., 2021). In clinical practice, BTC is categorized into intrahepatic cholangiocarcinoma (iCCA), extrahepatic cholangiocarcinoma (eCCA), and gallbladder carcinoma (GBC) based on anatomical tumor localization, which accounts for 10%–20%, 20%–30%, and 50%–60% of BTC, respectively (Banales et al., 2020; Javle et al., 2022). In recent years, the mortality rate of iCCA has been rising, reaching approximately 1–2 deaths per 100,000 population, thereby contributing significantly to the overall BTC mortality (Bertuccio et al., 2019). Unfortunately, iCCA typically presents symptoms only in its late stages, rendering surgical intervention impractical for achieving curative outcomes; moreover, the recurrence rate exceeds 75% within 2 years post-surgery (Z et al., 2018). Consequently, palliative systemic chemotherapy remains the standard first-line treatment. However, due to the high heterogeneity of iCCA, patients often develop resistance to standard therapies rapidly, leading to a median survival rate of only 6 months (Kang et al., 2022).

The genetic landscape of BTC has been extensively analyzed, revealing that nearly 40% of patients harbor potentially targetable genetic alterations (Harding et al., 2023). Among these, iCCA harbors more actionable pathogenic variants compared to eCCA and GBC, with common pathogenic variants including *IDH1/2* (15%), *BAP1* (11%), and *FGFR2* alterations (10%) (Goyal et al., 2021; Deiana et al., 2024). For the high-frequency pathological pathogenic variants, several targeted drugs have been approved by the FDA for follow-up treatment, including pemigatinib (Abou-Alfa et al., 2020a), futibatinib (Goyal et al., 2023), and infigratinib (Javle et al., 2021) for FGFR2 fusions, entrectinib for NTRK fusions (Doebele et al., 2020), as well as ivosidenib for IDH1 pathogenic variants (Abou-Alfa et al., 2020b). These targeted therapies offer longer-lasting benefits compared to traditional chemotherapy. Consequently, for patients with advanced iCCA who have failed first-line treatment, the development of individualized treatment plans based on molecular profiling results appears promising. However, the patients carrying BRCA pathogenic variants is relatively small, comprising only 4% of iCCA cases, leading to inconclusive evidence regarding the efficacy of targeted therapy with poly adenosine diphosphate-ribose polymerase inhibitors (PARPi) for these patients (Deiana et al., 2024). Herein, we present the first case of a patient with advanced ICCA harboring dual somatic homologous recombination repair (HRR) gene pathogenic variants (*BRCA1* and *PALB2*) who achieved a partial response following olaparib salvage treatment.

## Case presentation

**FIGURE 1:**
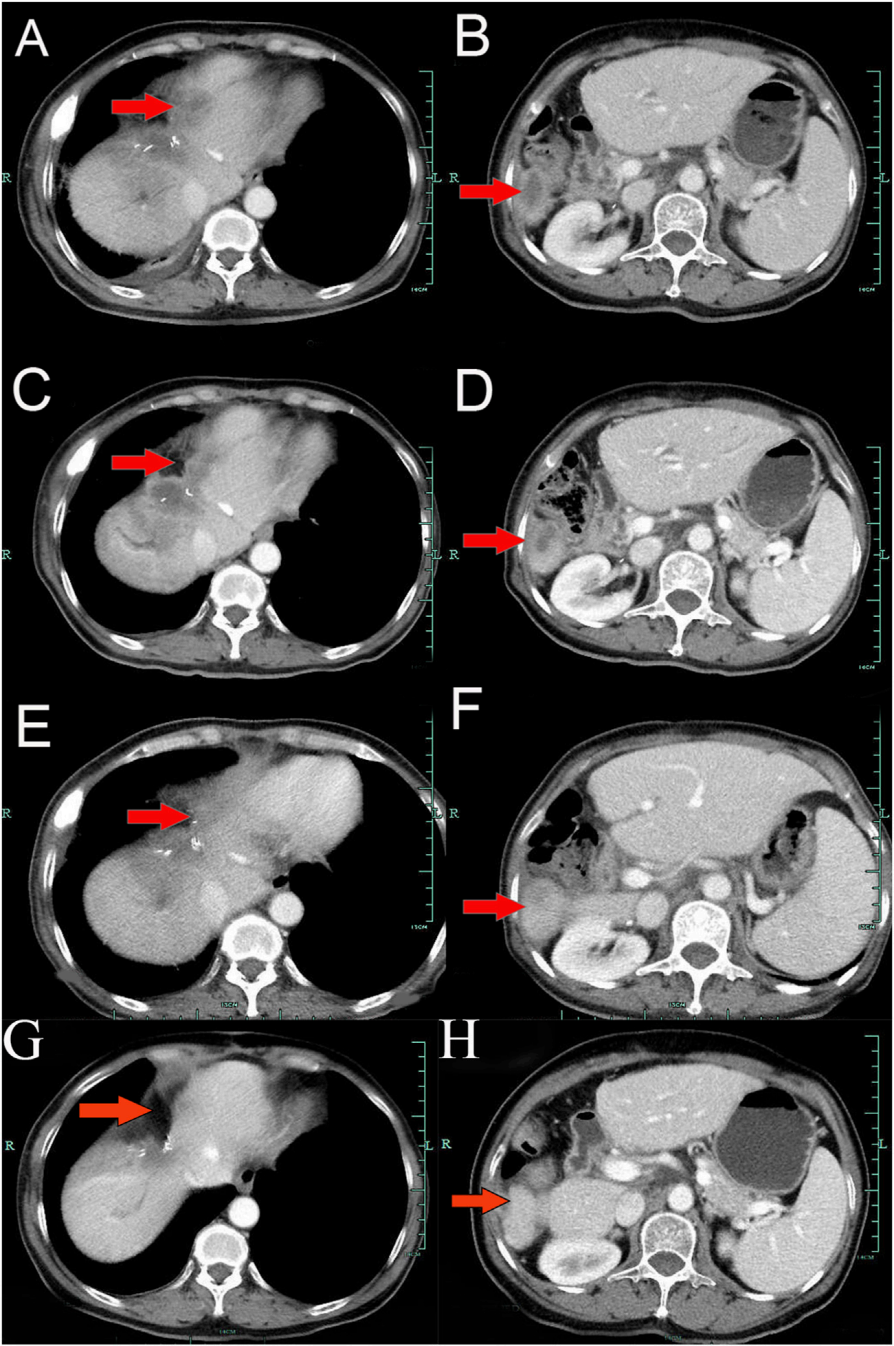
Variations of occupation on Liver areas by CT images during the treatment (red arrowheads). **(A,B)**, March 2024. **(C,D)**, April 2024. **(E,F)**, June 2024. **(G,H)**, October 2024.

**FIGURE 2:**
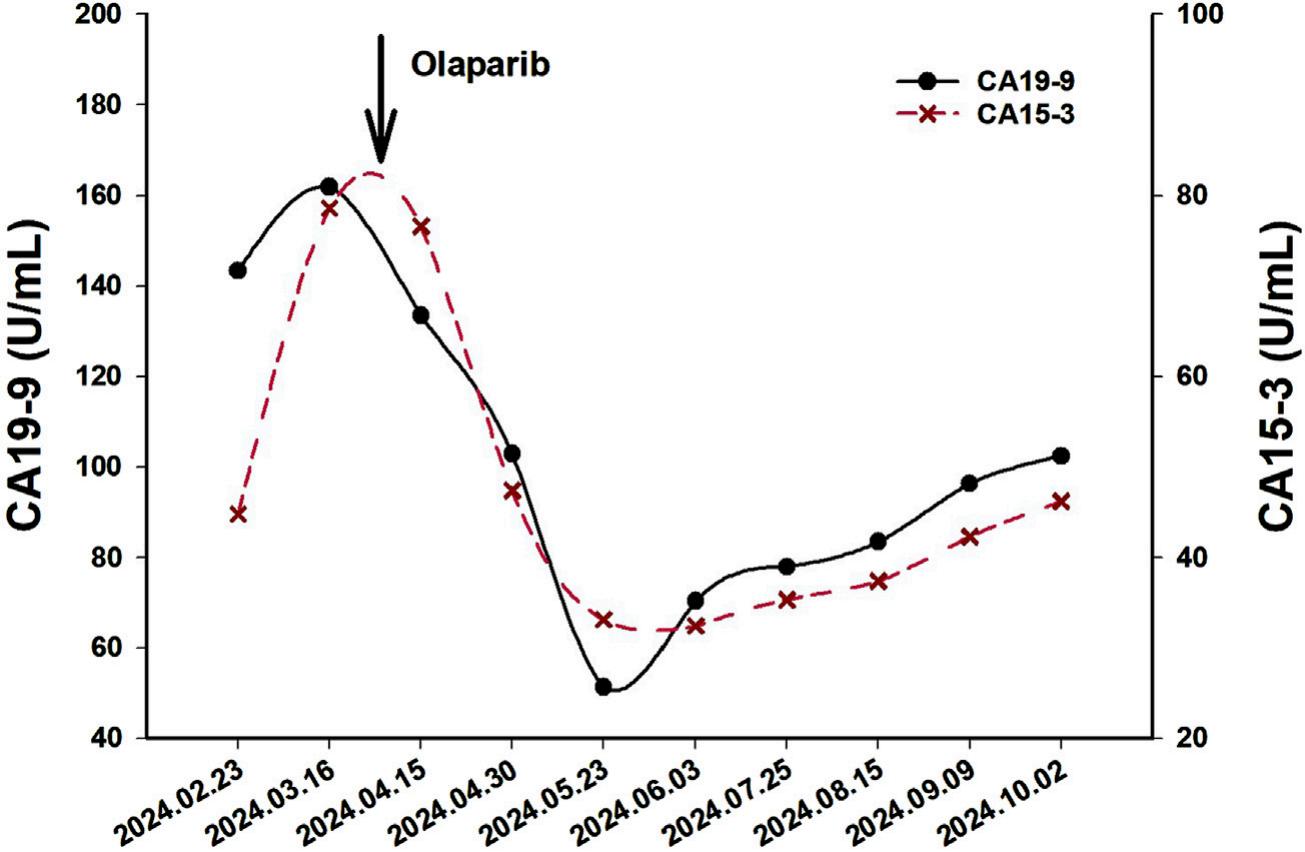
The variations of tumor marker CA19-9 (normal range 0–37 U/mL) and CA15-3 (normal range 0–25 U/mL) from February 23rd to 3 June 2024.

**FIGURE 3:**
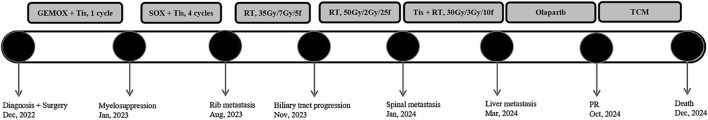
The overview of the approaches to diagnosis, and treatment regimens for each regimen. Abbreviations: GEMOX, Gemcitabine + Oxaliplatin; Tis, Tislelizumab; RT, Radiotherapy; SOX, S-1 +; Oxaliplatin; PR, Partial Response; TCM, Traditional Chinese Medicine.

A 62-year-old Chinese woman was admitted to Quzhou People’s Hospital on 24 December 2022, due to paroxysmal pain in the right upper abdomen for nearly 1 month. She had no history of smoking, alcohol use, significant comorbidities, or a family history of cancer. The next day, a contrast-enhanced computed tomography (CT) scan of the abdomen revealed a mass-like low-density shadow measuring approximately 6.1 × 4.9 cm in the right hepatic lobe of the liver, accompanied by mild dilation of the distal intrahepatic bile duct. A chest CT and an enhanced magnetic resonance imaging (MRI) of the brain showed no evidence of metastasis. Subsequently, the patient underwent an extended anatomical middle hepatic lobectomy (including segment IV, segment V, segment VIII, and the paracaval portion of the caudate lobe), combined with resection of the first and second hepatic portal vessels and biliary stricture reconstruction (partial resection and reshaping of the right anterior and posterior bile ducts), as well as lymphadenectomy of the hepatoduodenal ligament on 29 December 2022. The surgery was successfully completed, achieving an R0 resection with negative margins. Postoperative pathological and immunohistochemical analysis confirmed the diagnosis of moderately differentiated cholangiocarcinoma. Immunohistochemistry findings were as follows: CK7 (positive), CK19 (positive), CK20 (positive), CK18 (positive), Hepatocyte (negative), AFP (negative), GPC-3 (negative), CD34 (negative), CD10 (focally positive), CDX-2 (negative); Ki-67 (positive, 60%), and PD-L1 expression of <1%. The results of the next-generation sequencing (NGS) were as follows: *BRCA1* (exon 10, p.Q1111fs, VAF: 19.7%), *PALB2* (exon 9, p.E956*, VAF: 19.8%), and *TP53* (exon 5, p.Q165fs, VAF: 33.6%). Based on these findings, a diagnosis of cholangiocarcinoma was made and staged as T4NxM0 according to the 8th edition of the AJCC. The patient received the first postoperative immunotherapy combined with chemotherapy on 28 January 2023, which was tislelizumab (IV 200 mg) + gemcitabine (IV 800 mg on DAY 1/600 mg DAY 8) + oxaliplatin (IV 100 mg day 1). However, due to significant bone marrow suppression, the patient was subsequently treated with four cycles of tislelizumab (IV 200 mg on day 1, every 14 days), S-1 (IV 400 mg DAY2-15, every 14 days), oxaliplatin (IV 100 mg on day 1, every 14 days). Due to right-sided chest pain for 1 week, a repeated chest CT scan was performed on 8 August 2023. The results revealed the right seventh rib exhibited thickening and increased density, accompanied by swelling of the surrounding soft tissue. The patient subsequently underwent stereotactic body radiotherapy (SBRT) in this area, with a prescribed dose of 35 Gy in 5 fractions (7 Gy per fraction). The following abdominal CT scan on 11 November 2023, revealed mild dilatation of the intrahepatic bile duct, after which the patient received a radiotherapy regimen of 50Gy/2Gy/25f. Due to frequent low back pain, the patient underwent a lumbar spine MRI on 23 January 2024. The MRI revealed multiple patchy shadows in thoracic vertebrae 1, thoracic vertebrae 6–11, and the lumbar vertebrae. Following this, he received a single dose of tislelizumab (200 mg) and a follow-up SBRT treatment regimen of 30 Gy delivered in 3 Gy fractions over 10 sessions. Unfortunately, abdominal CT on 15 March 2024, revealed multiple new metastatic clusters (Figures 1A,B). Given the NGS result and the patient’s ECOG performance status of 2, as well as his strong desire to continue active treatment, we initiated salvage therapy with olaparib, an oral PARP inhibitor targeting BRCA, administered at 150 mg twice daily. Subsequent abdominal CT scans conducted on April 30 (Figures 1C,D)and 4 June 2024 (Figures 1E,F), demonstrated tumor regression, achieving a partial response (PR) according to the RECIST 1.1 criteria. The patient maintained PR without radiographic progression until treatment discontinuation in October 2024 (Figures 1G,H), when therapy was switched to traditional Chinese medicine due to financial limitations. Importantly, the patient tolerated olaparib well, with no treatment-related adverse events reported throughout the course of therapy. Concurrently, notable reductions in tumor markers CA199 and CA153 were observed during olaparib therapy (Figure 2), along with improvement in the patient’s performance status. Unfortunately, he passed away in December 2024 from type II respiratory failure secondary to pneumonia. A summary of the diagnostic approach, treatment regimens, and changes in targeted lesions is presented in Figure 3.

## Discussion

In this report, we presented a case of a patient with advanced ICCA harboring dual somatic HRR gene pathogenic variants, specifically *BRCA1* and *PALB2*, who achieved PR lasting approximately 7 months following salvage treatment with olaparib. Given the notable efficacy observed and thought-provoking nature of this case, we encourage future studies to focus more on *BRCA*, *PALB2*, and other HRR pathogenic variants in the BTC population, to broaden the effective targets of PARPi for BTC patients and clarify the characteristics of the beneficial subgroup.

HRR is a critical pathway in the DNA damage response, primarily responsible for repairing DNA double-strand breaks to maintain genomic stability (Qian et al., 2024). *BRCA1* and *BRCA2* are essential genes involved in HRR, alongside several other important genes, such as *PALB2*, *ATM*, and *BRIP1* (Ricci et al., 2020). Pathogenic variants in these genes can result in homologous recombination deficiency (HRD) and heightened genetic instability, thereby facilitating the occurrence and progression of various cancers, including breast, ovarian, prostate, and pancreatic cancer. Olaparib, the first FDA-approved PARPi, specifically targets the PARP enzyme, resulting in the accumulation of DNA damage and subsequent apoptosis of cancer cells with HRR gene pathogenic variants, a mechanism known as synthetic lethality. Additionally, current studies have also shown that *BRCA* gene pathogenic variants are the best clinical biomarkers for response to PARPi among HRR gene pathogenic variants (Wagener-Ryczek et al., 2021).

The current research on olaparib focuses on expanding its therapeutic applications to additional tumor types that may benefit from this treatment and explore other pathological targets. Notably, a deeper understanding of PARPi has revealed that in addition to its efficacy against conventional tumors with *BRCA* pathogenic variants, cancer patients with *PALB2* pathogenic variants also appear to derive benefit from this therapy. For example, olaparib can significantly enhance progression-free survival (PFS) in patients with metastatic castration-resistant prostate cancer who carry HRR gene alterations, including *PALB2* (de Bono et al., 2020). Additionally, in a Phase II study investigating the efficacy of maintenance rucaparib in patients with platinum-sensitive advanced pancreatic cancer harboring pathogenic germline or somatic variants, two PR and one complete response (CR) were observed in six patients with germline *PALB2* pathogenic variants (gPALB2m) following rucaparib treatment (Reiss et al., 2021). In a Phase II study of olaparib for treating metastatic breast cancer associated with HRR gene pathogenic variants, 9 out of the 11 patients with *gPALB2m* achieved PR, while 2 patients exhibited stable disease (SD) (Tung et al., 2020). Notably, this study also included two patients with somatic *PALB2* pathogenic variants (sPALB2m), both of whom achieved SD after olaparib treatment. Simultaneously, the updated ACMG guideline highlights that *PALB2* variants confer breast and ovarian cancer risks comparable to *BRCA1/2* and recommends reporting these variants as secondary findings in clinical exome and genome sequencing (Miller et al., 2021). Consequently, these findings suggest that *PALB2* might become the next key biomarker for PARPi after *BRCA*.

With the increasing number of studies reporting on BTC associated with *BRCA* pathogenic variants in recent years, targeted therapy has assumed a pivotal role in treatment. This development is encouraging for BTC patients with limited treatment options in the late stages of the disease and indicates that BTC may also benefit from PARPi. A search in Medline and Embase using the keywords “*BRCA*,” “*BRCA1*,” “*BRCA2*,” “*PALB2*,” “cholangiocarcinoma,” “Biliary tract cancer,” and “gallbladder carcinoma” identified 22 studies with 56 BTC cases (Table 1), the largest and most recent review to date, covering cases published between 2015 and 2024 (Zhang et al., 2021; Prabhakar et al., 2024; Smith et al., 2023; Almujarkesh et al., 2023; Yang et al., 2023; Costa et al., 2023; Kim et al., 2023; Zhou et al., 2022; Su et al., 2021; Xie et al., 2016; Thavaneswaran et al., 2023; Joris et al., 2023; He et al., 2022; Yamashita et al., 2023; Dong et al., 2023; Rimini et al., 2022; Li et al., 2021; Xiong et al., 2020; Cheng et al., 2018; Sharma et al., 2016; Chen et al., 2024; Golan et al., 2017). As shown in Table 1, the patient’s age ranged from 39 to 79 years, and a male-to-female ratio of 24:9, of which 32 cases had unclear age and 23 had unclear gender. The pathogenic variant distribution included 12 *sBRCA1m*, 10 *sBRCA2m*, 5 *gBRCA1m*, 7 *gBRCA1m*, 1 *sPALB2m*, 3 structural variants *BRCA*, 1 double *BRCA1/2* pathogenic variant, 1 germline and somatic *BRCA2* pathogenic variant, and 15 *BRCA* pathogenic variants for which detailed information was not available. Most patients received PARPi monotherapy or combined immunotherapy, while fewer received platinum-based chemotherapy. Interestingly, PD-1 inhibitors and PARPi appeared in the treatment of 4 of the five CR patients, with 2 of them receiving a combination of Pembrolizumab and olaparib (Smith et al., 2023; Zhou et al., 2022; He et al., 2022; Dong et al., 2023; Xiong et al., 2020). Additionally, Nicholas Prabhakar also reported a case of a CCA patient with *BRCA1* and *BRCA2* co-pathogenic variants who achieved a 15-month PFS after receiving olaparib treatment (Prabhakar et al., 2024). Moreover, Margherita Rimini reported a case of an ICCA patient harboring *sPALB2m* who achieved a PFS of 3.2 months following PARPi monotherapy (Rimini et al., 2022). To further illustrate the use of PARP inhibitors, Table 2 summarizes key Phase III trials of olaparib in other BRCA/HRD-driven cancers—including HRD-positive breast cancer (OLYMPiAD trial) (Tutt et al., 2021), the POLO trial in pancreatic cancer (Gola et al., 2019; Kindler et al., 2022), and BRCA1/2-mutated prostate cancer (PROfound trial) (de Bono et al., 2020)—which demonstrated significant improvements in both progression-free survival and overall survival.

**TABLE 1:** Clinical characteristics, treatment and outcomes of biliary tract cancer.

Author (year)	Gender	Age	Mutation targets	Somatic (SM) and/or germline (GL) BRCA mutations	Surgery	Primary site	Treatment	Sample size	Outcome (months)
Prabhakar et al. (2024)	F	71	Both BRCA1/2	SM BRCA1/2 & GM BRCA2	Yes	CCA	Olaparib	1	PFS:15
Chen et al. (2024)	F	56	BRCA2	SM	No	GBC	Olaparib & Durvalumab	1	PR; PFS:29
Smith et al. (2023)	M	79	BRCA1	SM	No	ECCA	Talazoparib	1	CR; PFS:36+
Almujarkesh et al. (2023)	F	50	BRCA: NA	GM	Yes	ECCA	Platinum-based chemotherapy	1	NR
Joris et al. (2023)	F1,M6	NA	BRCA1	GM:2; SM:5	NA	GBC:6; CCA:1	Olaparib	7	SD:5; PR:1; PD:1
Joris et al. (2023)	M	NA	BRCA2	SM:1; GM:1	NA	GBC	Olaparib	2	PD
Thavaneswaran et al. (2023)	NA	NA	BRCA2	NA	NA	CCA	Olaparib& Durvalumab	1	PR; PFS:6+
Thavaneswaran et al. (2023)	NA	NA	BRCA2	NA	NA	GBC	Olaparib & Durvalumab	1	PR; PFS:36+
Yang et al. (2023)	M	52	BRCA 1	GM	No	GBC	Olaparib	1	PR; PFS:6+
Yamashita et al. (2023)	M	73	BRCA1	GM	No	ICCA	Gemcitabine & Cisplatin	1	PR; PFS:4+
Dong et al. (2023)	NA	NA	BRCA2	NA	Yes	GBC	GemOX & Sintilimab & Olaparib	1	CR; PFS:2+
Costa et al. (2023)	M	53	BRCA2	GM	No	ECCA	Olaparib	1	PR; PFS:36+
Kim et al. (2023)	M6; F3	61	BRCA1:3 BRCA2:5 Both:1	NA	NA	GBC:4 ICCA:5	Platinum-based chemotherapy	9	mPFS:6.7 mOS:10.6
He et al. (2022)	M	67	BRCA1	NA	No	ICCA	Camrelizumab& Paclitaxel	1	CR; PFS:32+
Rimini et al. (2022)	NA	NA	BRCA2	SM	Yes	ICCA	PARPi	1	PFS:0.5
Rimini et al. (2022)	NA	NA	PALB2	SM	Yes	ICCA	PARPi	1	PFS:3.2
Zhou et al. (2022)	M	53	BRCA2	Both	Yes	ICCA	Pembrolizumab& Olaparib	1	CR; PFS:15+
Li et al. (2021)	M	68	BRCA2	SM	Yes	ECCA	Olaparib	1	SD; PFS:10+
Su et al. (2021)	M	71	BRCA2	SM	No	CCA	Olaparib	1	PR; PFS:7+
Zhang et al. (2021)	M	72	BRCA2	NA	Yes	CCA	Olaparib	1	PR
Xiong et al. (2020)	M	43	BRCA1	SM	Yes	ICCA	Olaparib & Pembrolizumab	1	CR:PFS:9+
Cheng et al. (2018)	F	64	BRCA2	GM	No	ICCA	Olaparib	1	PR; PFS:9+
Golan et al. (2017)	NA	NA	BRCA1:1 BRCA2:3	SM BRCA2:1 GM BRCA1/2:1/2	3	NA	PARPi	4	mOS:32.91
Golan et al. (2017)	NA	NA	BRCA1:7 BRCA2:7	SM BRCA1/2:5/4 GM BRCA1/2:0/2 SV BRCA1/2:2/1	7	NA	No PARPi	14	mOS:25.96
Sharma et al. (2016)	F	39	BRCA2	GM	No	CCA	Gemcitabine & cisplatin	1	PR; PFS:4+

PFS, Progression-Free Survival; mPFS, median progression-free survival; PARPi, poly adenosine diphosphate-ribose polymerase inhibitors; ICCA, intrahepatic cholangiocarcinoma; ECCA, extrahepatic cholangiocarcinoma; GBC, gallbladder carcinoma; CCA, cholangiocarcinoma; PR, partial response; SD, stable disease; PD, progressive disease; NR, not reported; NA: not applicable; OS, overall survival;mOS, median overall survival; CR, complete response; M, male; F, female; SV, structural variant; GM, germline mutation; SM, somatic mutation.

**TABLE 2:** Clinical characteristics, treatment regimens, and key outcomes of Phase III trials of olaparib in BRCA/HRD-driven cancers: breast cancer (OLYMPiAD trial), pancreatic cancer (POLO trial), and prostate cancer (PROfound trial).

Author (year)	Gender	Age (median range)	Primary site	Treatment	Sample size	Outcome (months)
Tutt et al. (2021)	F	42 (36–49) vs. 43 (36–50)	Breast cancer	Olaparib vs. Placebo	921 vs. 915	3y-IDFS: 85.9% vs. 77.1%, HR = 0.58; 3y-DDFS: 87.5% vs. 80.4%, HR = 0.57
Gola et al. (2019), Kindler et al. (2022)	F: 70 M: 84	57 (37–84) vs. 57 (36–75)	Pancreatic cancer	Olaparib vs. Placebo	92 vs. 62	mPFS: 7.4 vs. 3.8, HR = 0.53; mOS: 19 vs. 19.2, HR = 0.83
Johann de Bono (2020)	M	68 (47–86) vs. 67 (49–86)	Prostate cancer	Olaparib vs. enzalutamide or abiraterone	162 vs. 83	mPFS: 7.4 vs. 3.6, HR = 0.34; mOS:18.5 vs. 15.1, HR = 0.64;

mPFS, median progression-free survival; mOS, median overall survival; 3y-IDFS, 3-year invasive disease– free survival; 3y-DDFS, 3-year distant disease–free survival; M, male; F, female.

As illustrated in Figure 3, this advanced patient with dual HRR pathogenic variants exhibited inadequate disease control following systemic chemotherapy and radiotherapy. We hypothesize that the presence of dual HRR pathogenic variants may exacerbate deficiencies in DNA repair function, which could enhance tumor heterogeneity, diminish tumor sensitivity to conventional treatments, and accelerate disease progression (Kiwerska and Szyfter, 2019). Concurrently, the dual pathogenic variants in HRD tumor cells may increase reliance on the PARP-mediated DNA repair mechanism, thereby enhancing the response to PARPi and ultimately resulting in PR. The above literature review indicates that both somatic and germline pathogenic variants of *BRCA1/2* can respond positively to PARPi. Furthermore, a patient with a somatic *PALB2* pathogenic variant exhibited similar benefits, aligning closely with the findings in this study. Simultaneously, a recent research conducted by Japanese scientists has demonstrated that pathogenic variants of *PALB2* are marginally enriched in BTC, suggesting that the *PALB2* gene may serve as a potential risk factor. Consequently, we propose two key perspectives: firstly, the BTC population with dual HRR pathogenic variants, one of which includes *BRCA*, may represent an advantageous cohort for olaparib treatment; secondly, beyond *BRCA* pathogenic variant, *PALB2* pathogenic variant may emerge as the next clinical predictive target for PARPi in the BTC population.

This case report presents several limitations. Firstly, the generalizability of its results may be restricted, and the observed outcomes might be coincidental due to the nature of case reports. Secondly, the HRD evaluation was not conducted on this patient because there is no consensus on HRD scoring criteria. Additionally, the criterion for parameters varies across different detection methods, which further contributes to the variability and uncertainty of the findings. Furthermore, the rapid progression of the patient’s disease, coupled with multiple tumor metastases, has severely limited subsequent treatment options. Given the few reported cases of olaparib’s efficacy in treating BTC with *BRCA* pathogenic variants and the patient’s strong desire for survival, olaparib salvage treatment was ultimately pursued, resulting in partial remission. Lastly, the optimal treatment strategy for BTC with *BRCA* pathogenic variants remains to be established.

In summary, this study reported a case of ICCA harboring *BRCA1* and *PALB2* pathogenic variants, which exhibited rapid progression following a combination of immunotherapy, chemotherapy, and radiotherapy, but achieved partial response after receiving olaparib as salvage treatment. Furthermore, a systematic summary and analysis of reported BTC studies involving *BRCA* pathogenic variants suggest that such patients may benefit from olaparib. Currently, related clinical trials are underway, which may provide new treatment options for the BTC population, characterized by limited therapeutic alternatives and poor prognosis, and further expand the range of tumors amenable to olaparib treatment. Additionally, the *PALB2* pathogenic variant may emerge as a significant clinical predictive target for PARPi in the BTC population.

## Data Availability

The raw data supporting the conclusions of this article will be made available by the authors, without undue reservation.
